# Personalized or Precision Medicine? The Example of Cystic Fibrosis

**DOI:** 10.3389/fphar.2017.00390

**Published:** 2017-06-20

**Authors:** Fernando A. L. Marson, Carmen S. Bertuzzo, José D. Ribeiro

**Affiliations:** ^1^Department of Medical Genetics, Faculty of Medical Sciences, State University of CampinasCampinas, Brazil; ^2^Department of Pediatrics, Faculty of Medical Sciences, State University of CampinasCampinas, Brazil

**Keywords:** *CFTR*, genotype, gene-therapy, lung disease, phenotype, variability

## Abstract

The advent of the knowledge on human genetics, by the identification of disease-associated variants, culminated in the understanding of human variability. With the genetic knowledge, the specificity of the clinical phenotype and the drug response of each individual were understood. Using the cystic fibrosis (CF) as an example, the new terms that emerged such as personalized medicine and precision medicine can be characterized. The genetic knowledge in CF is broad and the presence of a monogenic disease caused by mutations in the *CFTR* gene enables the phenotype–genotype association studies (including the response to drugs), considering the wide clinical and laboratory spectrum dependent on the mutual action of genotype, environment, and lifestyle. Regarding the CF disease, personalized medicine is the treatment directed at the symptoms, and this treatment is adjusted depending on the patient’s phenotype. However, more recently, the term precision medicine began to be widely used, although its correct application and understanding are still vague and poorly characterized. In precision medicine, we understand the individual as a response to the interrelation between environment, lifestyle, and genetic factors, which enabled the advent of new therapeutic models, such as conventional drugs adjustment by individual patient dosage and drug type and response, development of new drugs (read through, broker, enhancer, stabilizer, and amplifier compounds), genome editing by homologous recombination, zinc finger nucleases, TALEN (transcription activator-like effector nuclease), CRISPR-Cas9 (clustered regularly interspaced short palindromic repeats-CRISPR-associated endonuclease 9), and gene therapy. Thus, we introduced the terms personalized medicine and precision medicine based on the CF.

## Introduction

Currently, numerous and significant advances have been achieved on the pathophysiological and genetic knowledge of numerous diseases. These advances are associated with the advent of new technologies related to diagnosis, treatment, and reduction of costs from genetic studies and implementation of new management approaches, resulting mainly from multicenter studies and meta-analyses. These studies have shown significant population variability and enabled the implementation of databases with numerous genetic variants, including mutations and polymorphisms (Bianco et al., 2013; Xu et al., 2016).

**FIGURE 1:**
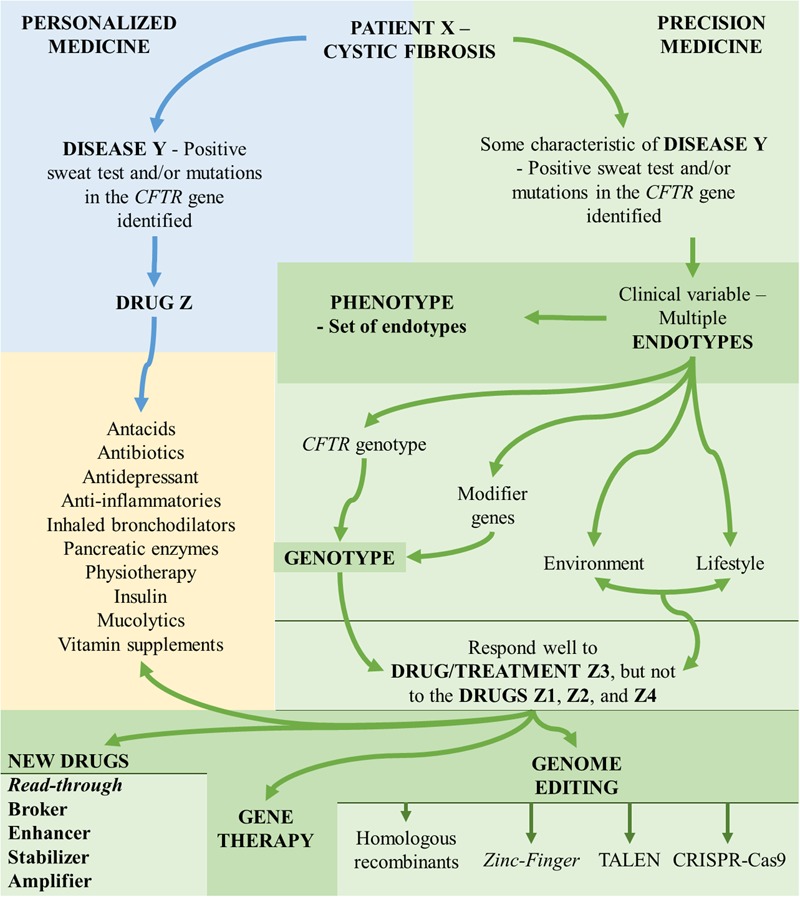
Aspects associated with the understanding of personalized medicine and precision medicine. *CFTR*, cystic fibrosis transmembrane regulator; TALEN, transcription activator-like effector nuclease; CRISP-Cas9, clustered regularly interspaced short palindromic repeats-associated endonuclease 9.

The importance of these skills to the internist, researchers, geneticist, and other health professionals is that, increasingly, every disease is demonstrated as a complex of many diseases, regarding the symptoms, divided into numerous genotypes, phenotypes, and endotypes (**Figure 1**).

The genetic part that characterizes the diseases has been demonstrated in numerous studies, even in diseases that are true public health problems, such as asthma and tuberculosis, among the diseases of the respiratory tract. With the increase of genetic knowledge, it is essential to analyze the cost-benefit, cost-effectiveness, and cost-utility (Scott, 2016), application of ethical rigor necessary to identify mutations and variants in diagnostic and screening tests, especially considering the pediatric age group and the amount of information that can be generated with collection of genetic material and its laboratory analysis, as well as its application (Botkin, 2016).

In the study of genetic variants, the determination of genes, their polymorphisms and mutations, which are associated with certain diseases, and their variability is constant, including the CF (OMIM: #219700). In recent decades, the implementation of genetic knowledge on CF occurred, mostly, by obtaining and using new methods to identify genetic variants (Ziętkiewicz et al., 2014; Baker et al., 2016; Lim et al., 2016; Lundman et al., 2016; Straniero et al., 2016; Ratkiewicz et al., 2017).

## Cystic Fibrosis Disease

**FIGURE 2:**
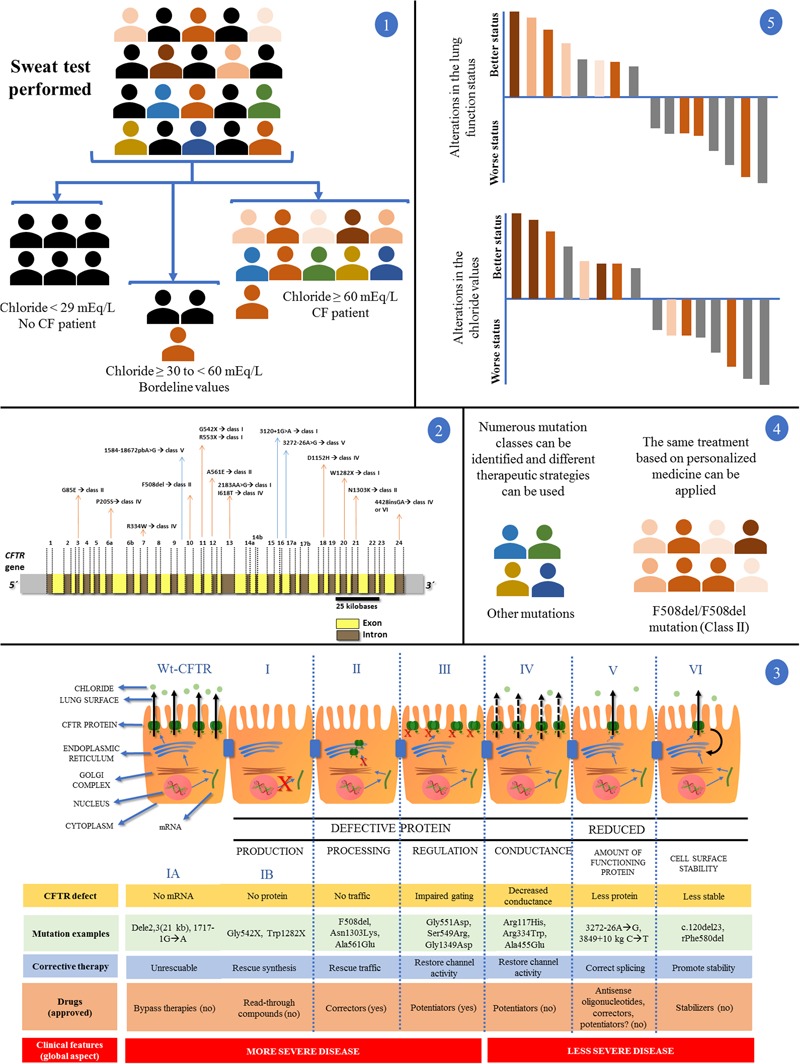
Clinical and laboratory response achieved with the introduction of personalized medicine and precision medicine in CF regarding disease diagnosis by sweat test and *CFTR* (cystic fibrosis transmembrane regulator) mutation screening. **(1)** In a population, patients with suspected CF underwent the sweat test. Chloride values equal to/greater than 60 mEq/L are the gold standard for CF diagnosis. Chloride values smaller than 30 mEq/L are related with normal subjects for CF diagnosis. Regarding chloride values between 30 and 60 mEq/L, nearly 5% of patients with CF show positive screening for the *CFTR* mutations. After the test, patients can be divided into two groups: (i) normal and borderline values for chloride test without CF diagnosis (black color); (ii) patients with CF (color symbols + some patients with CF and borderline values in the sweat test). **(2)** Patients with CF, in some centers around the world, can undergo the *CFTR* genetic screening. The figure shows the *CFTR* gene containing introns (yellow) and exons (brown). Some mutations and their locations were shown. The main *CFTR* mutation is the F508del, which affects most patients with CF. **(3)** First is shown the CFTR protein mechanisms from transcription to cell surface anchoring and function. Defective CFTR protein is observed followed by class I, II, III, and IV. Reduced CFTR protein function is observed followed by class V and VI. Class I: No production by no transcription (IA, no mRNA; IB, no protein). Class II: CFTR processing error. CFTR protein is degraded in endoplasmic reticulum. Class III: CFTR protein regulation with defect. *CFTR* mutations related with the R domain expression. Class IV: conduction is altered by mutations related by membrane-spanning domains. Class V: reduced amount of CFTR protein at cell surface – however, normal activity is present. Class VI: low CFTR function stability. CFTR, cystic fibrosis transmembrane regulator; red (X) indicates absence of CFTR protein by premature stop codon (class I), absence of CFTR protein at cell surface (class II) or absence of CFTR function (class III); black continuous arrows indicate normal chloride transport; black no continuous arrows indicate residual chloride transport. The number of CFTR protein at cell surface is related with *CFTR* gene expression. Moreover, there is a short description regarding the CFTR defect, mutation examples, corrective therapy, drugs, and clinical features. **(4)** After the *CFTR* genetic screening we can divide the patients with CF according to *CFTR* mutation classes, additionally, in each class we can perform the direct treatment. The figure shows a patient group with the F508del/F508del genotype and another group with numerous genotypes. By personalized medicine, we can treat the patients regarding the *CFTR* mutation class (in theory), and, in some cases, we can develop the drug for specific *CFTR* mutations. **(5)** Regarding only patients with CF and F508del/F508del genotype, after the use of the new drugs (i.e., VX809-VX770), we can achieve patients with CF plus positive response and negative response using or not the drugs comparing the results with placebo (gray data). In this case, the F508del/F508del genotype (orange data) has a wide variability in the drug response dependent on the mutual action of genotype, environment, and lifestyle (intensity of the orange color). All the data can be observed in details in Boyle et al. (2014), Marson et al. (2015, 2016), De Boeck and Amaral (2016), and Farrell et al. (2017).

The CF is a monogenic, autosomal, and recessive disease, with highly variable and complex clinical expression that had the identification of its causal gene in 1989, the known *CFTR* gene in chromosome 7q31.2 (*CFTR*) (Chang and Zabner, 2015; Castellani and Assael, 2016). From that date, numerous studies have been published and led to the identification of approximately 2,000 mutations, only in that gene (Cystic Fibrosis Mutation Database, accessed on 05/01/2017). The CF diagnosis and diversity according to the *CFTR* mutation status can be observed in **Figure 2**.

The clinical manifestations in CF are complex, and even decades after the description of the disease and identification of the *CFTR* gene, not every spectrum of the disease is known or even understood, including the interaction between socioeconomic status and health outcomes (Castellani and Assael, 2016; Oates and Schechter, 2016; Brown et al., 2017). In addition, the diagnosis and clinical approach in CF are crucial to the understanding of the disease, targeting of the treatment, and follow-up of the progression of characteristic phenotypes. This fact is useful and essential in all chronic diseases.

In CF, as well as in other diseases, the genetic knowledge involved determines the causal basis of the disease, provides the genetic counseling and the possibility of understanding the clinical variability (phenotypes and endotypes), as well as the response to the treatment and new therapeutic modalities. In recent years, CF has been a model for numerous studies (approximately 45,000 publications in PubMed), and, in most of them, genetics and management go hand in hand (Marson et al., 2015), and the therapy, most of the time, is based on the treatment of symptoms and follow-up of patients (Castellani and Assael, 2016).

On the other hand, based on the knowledge gained from the molecular study, the implementation of the personalized medicine occurred, mainly, for the new read-through drugs, enhancers, CFTR protein stabilizers and amplifier compounds (Boyle and De Boeck, 2013; Ikpa et al., 2014; Marson et al., 2015; Quon and Wilcox, 2015; Corvol et al., 2016; De Boeck and Amaral, 2016; Lopes-Pacheco, 2016; Schmidt et al., 2016; Schneider et al., 2016; Spielberg and Clancy, 2016; Liang et al., 2017; Rafeeq and Murad, 2017) (**Figures 1, 2**).

## Personalized Medicine – the Example of Cystic Fibrosis

Personalized medicine is a term used for the treatment focusing on the patients based on their individual clinical characterization, considering the diversity of symptoms, severity, and genetic traits. Thus, personalized medicine is performed in CF, and in many other diseases, based on the patients’ symptoms. A classic example is the use of supplementation of digestive enzymes in CF. The dose is adjusted, not only because of the patient’s physiological characteristics, but considering the response to the enzyme, volume of food ingested, type of food ingested, number of meals, body mass gain, growth rate, and type of enzyme used (Castellani and Assael, 2016; Ozen Alahdab and Duman, 2016; Sathe and Freeman, 2016). The same occurs for other clinical symptoms in CF, such as chronic pulmonary infection (Castellani and Assael, 2016).

However, today, perspectives and directions have been questioned and implemented to seek mechanisms to treat the disease and not the clinical signs and symptoms. In the case of pulmonary disease in CF, the signs and symptoms caused by the gravity associated with chronic inflammation and infection of lungs with resistance to antibiotics, progressive during the patients’ lives, is a condition that requires further study (Rutter et al., 2016; Samson et al., 2016).

Thus, we cannot characterize the personalized medicine as the medicine of the genomic era, but as the medicine that aims to treat the particularities of patients, often disregarding or simplifying the genetic nuances.

## Precision Medicine – an Innovation that Emerged

Overlapping the personalized medicine or a more complex model of understanding the phenotypic presentation, in 2015, the term precision medicine began to be used. The father of the precision medicine is researcher Archibald E. Garrod (1857–1936), who described the ubiquity of the individual variation, both in cases of diseases and in the identification of the human variability (Perlman and Govindaraju, 2016).

In precision medicine, the molecular information maximizes the accuracy with which the patients are categorized and treated, i.e., we have an endotype (or phenotypic variant) of a disease, which comes from a gene or group of genes and their interaction with the environmental factor and lifestyle (Perlman and Govindaraju, 2016) (**Figure 1**). In CF, we have multiple gravity phenotypes and clinical manifestations that are described and come from complex interactions between mutations in the *CFTR* gene, modifier genes, environment, and lifestyle (Marson et al., 2015) (**Figure 1**).

Among the concepts and definitions of precision medicine we have three aspects that must be considered: (i) traditional medicine should not be denied and must be recognized as the basis of precision medicine; (ii) precision medicine is not equal to simple convergence of new technologies – the relevant information for genomic knowledge requires effective integration with classical genetics, metabolomics, and clinical phenotypes (including symptoms and clinical signs, biochemical markers, and image and pathological characteristics, among others) to compose an individual and complete biological database, and contribute to diagnosis and treatment that are based on the patient’s individuality; (iii) precision medicine is not equal to a simple and individualized drug, but a medicine that combines standardization and individualization (Wang et al., 2016).

On the other hand, the implementation of precision medicine presents barriers that need to be overcome, such as: (i) regulation by governmental and medical entities, including the use of new ethical regulations; (ii) high cost for its implementation and for the pharmacological treatments obtained from the knowledge acquired; (iii) how to use and disclose information appropriately; (iv) how to approach and direct what will be done with patients who are part of the research projects before the status of the Government, the pharmaceutical industry and expectations of patients and/or family members; (v) analysis of bioinformatics that is still limited; (vi) the computational data analysis system needs to be better implemented; (vii) construction of prediction and interaction programs for clinical, laboratory, and genetic evaluation; (viii) large-scale sequencing, as well as punctual, also not available to all individuals who may benefit from the future of precision medicine (Biane et al., 2016; Breckenridge et al., 2016; Guo et al., 2016; Xue et al., 2016). Moreover, we should consider how the intervention may change the individual, especially when held in the first months and/or years of life (Breckenridge et al., 2016). The decision is not exclusively associated with the doctor, being dependent on a complex interpretation of data to ponder which drug will enable better performance in response to the problem within the biological system considered, which is unique for each individual (Biane et al., 2016).

Precision medicine, despite the use of complex techniques and data analysis, still follows the analysis model of reconstruction, based on the reductionist theory, showing difficulty in understanding the adaptive degree of the body regarding change, as well as the quest to control the precision of the method applied. Thus, we should seek a model to study and apply precision medicine at the holistic level, which is a barrier we will have to overcome for greater development in the application of the theory in the next years (Yuan, 2016).

## Precision Medicine – the Example of Cystic Fibrosis

Cystic fibrosis is one of the most important examples to describe the precision medicine. However, the problems found in CF for precision medicine treatment are complex to be solved, although CF is a monogenic disease. However, when dealing with other diseases, such as asthma, which is complex – resulting from the interaction of the environment with multiple genes (polygenic): each gene determines a small fraction in response to the drug used – the use of complete and complex tools that determine metabolic networks for polygenic modulation must be implemented and enables the use of precision medicine (Kersten and Koppelman, 2016) and/or the evaluation of phenotypes in common with other diseases, such as the CF, aiming at a possible interpolation of treatments, which would reduce the costs of treatment of CF and improve the treatment of asthma.

Researchers on CF offered us the publication of numerous studies, which enabled new prospects for the treatment of the disease, especially the pulmonary disease, with studies focusing on precision medicine (Boyle and De Boeck, 2013; Ikpa et al., 2014; Marson et al., 2015; Quon and Wilcox, 2015; Corvol et al., 2016 ; De Boeck and Amaral, 2016; Lopes-Pacheco, 2016; Schmidt et al., 2016; Schneider et al., 2016; Spielberg and Clancy, 2016). Thus, the CF is a model of precision medicine (Green, 2013) and, due to its potential as a study model (Martiniano et al., 2016), it has allowed, in some countries, the targeted treatment, as for example in the United States, where new drugs are used, based on genetic disorders, for approximately 50% of the patients with CF.

Despite the numerous studies on CF, there are still doubts about the applicability of precision medicine, then called personalized medicine, when we considered the read-through drugs, enhancers, stabilizers, and amplifiers (Balfour-Lynn, 2014; Bilton, 2014). Moreover, even in CF, which is a monogenic disease, we have a genetic and protein network that acts on drug responses and has direct action on drug response (Pankow et al., 2015) (**Figure 2**).

In CF, models of the *CFTR* molecular dynamics enabled the understanding of the interaction between the different effectors of the metabolic pathway that composes the CFTR protein expression and the evolution in the treatment and knowledge of the new drugs (Callebaut et al., 2016). However, progress is still required to obtain study models with simple and effective maintenance capacity, with general availability and universal applicability to the study of the patient’s disease and its multiple phenotypes. To this end, investments and persistence of pharmacotherapeutic studies and promptly executable genetic modification will be welcome (Martin, 2015; Mou et al., 2015). If on the one hand the difficulty of studying a monogenic disease is clear, we are also limited for other more complex diseases, such as asthma.

However, regardless of the precision or traditional medicine, we have to be aware of and participate in a holistic medicine, directed to the patient, not to the disease itself. It must be considered that various drugs interact with one another and with the body, and that we must seek the best way to achieve the balance between the individual and a good response to the treatment, maintaining and/or improving the quality and expectation of life (Jordan et al., 2016).

Studies on CF can be a source of knowledge about the treatment of other diseases, especially of chronic obstructive pulmonary diseases. One of these models can be assessed as to the response of drugs used in CF and also in other respiratory diseases, such as inhaled antibiotics, which has had its response evaluated in the personalized/precision medicine (Biller, 2015).

Studies of common phenotypes, particularly of clinical manifestations, of different diseases, allow knowledge development and cost reduction for precision medicine, and some of these studies are conducted in diseases with pulmonary phenotypes (Priyadharshini and Teran, 2016).

In addition to the drugs available by traditional and precision medicine, aforementioned, we have the possibility of gene therapy (which has as main barrier the layer of mucus in the lungs, preventing the transferring of genetic material) (Cooney et al., 2016; Duncan et al., 2016; Griesenbach et al., 2016; Steines et al., 2016) and genome editing by techniques such as homologous recombination, zinc finger nucleases, TALEN (Prather et al., 2013; Ramalingam et al., 2014; Camarasa and Gálvez, 2016; Suzuki et al., 2016), and CRISPR-Cas9 (Schwank et al., 2013; Bellec et al., 2015; Alapati and Morrisey, 2016), which are able to deal with the problems related with the mutations in the *CFTR* gene. However, we must be careful with expectations, considering what is real and what may just be fictitious (Alton et al., 2016).

All the information obtained from genetic studies has lead to the realization of a dream, which is the implementation of precision medicine in the healthcare network (Dzau and Ginsburg, 2016), which will culminate in the targeted treatment of monogenic diseases, as in the case of CF, and the treatment should be broad enough to “cover” all classes of mutations and/or specific mutations in the *CFTR* gene, with reduction of age for the beginning of drug use (Davies, 2015). All therapeutic approaches should result from the interaction between genetic and environmental factors and lifestyle. However, the question concerning the main problem that must be faced remains: Precision medicine: what is the price to be paid? (Ferkol and Quinton, 2015). Nowadays, there are high costs related with the genetic screening and the new treatments by precision medicine, with restricted availability for most CF patients.

Basic understanding of precision medicine and of the techniques employed, mainly in the area of genetics, will be extremely necessary for physicians, regardless of their specialty, as we advance quickly toward the genomics and precision medicine era. Based on a monogenic disease, such as the CF, we can describe the concepts of personalized medicine and precision medicine and aid in the dissemination and in the possibility of using personalized medicine and precision medicine.

In short, we simply described the aspects and concepts involved in both personalized and precision medicine, as well as the therapeutic possibilities that have emerged in CF by the knowledge of precision medicine. The data presented can be briefly observed in **Figure 1**, where there is characterization of the flowchart regarding CF variability and conceptualization of personalized medicine and precision medicine. Moreover, **Figure 2** shows an example regarding the clinical and laboratory response achieved with the introduction of personalized medicine and precision medicine and, in addition, we include a short flowchart regarding CF diagnosis, *CFTR* gene, and *CFTR* mutation classes.

## Future and Challenges in Personalized Medicine and Precision Medicine

Currently we have the availability of techniques for molecular analysis of numerous diseases. However, the development of drugs for various disorders is lacking, and in some cases (i.e., CF) in which the drug exists, there is lack of therapeutic efficacy. Another limiting factor is the high cost related to the diagnosis and application of precision medicine drugs. We must also consider the need to expand medical education for the new era of genetics, with broad knowledge of human genetic diversity and applicability in the treatment and follow-up of various diseases, including the CF disease.

## Conclusion

In recent years, many advances have been made in medical genetics, which led to the development of personalized medicine and precision medicine, making real a dream of many researchers, family members, and patients.

Personalized medicine is the treatment directed to the symptoms, and this treatment is adjusted depending on the patient’s phenotype. However, more recently, the term precision medicine began to be widely used although its correct application and understanding are still vague and poorly characterized. In precision medicine, we understand the individual as a response to the interrelation between environment, lifestyle, and genetic factors, which enabled the advent of new therapeutic models, such as conventional drugs adjustment by individual patient dosage and drug type and response, new drugs development (read through, broker, enhancer, stabilizer and amplifier compounds),genome editing by homologous recombination, zinc finger nucleases, TALEN, CRISPR-Cas9, and gene therapy. Thus, this mini review introduced the terms personalized medicine and precision medicine based on the CF.

Many of the problems related to the implementation of precision medicine could be solved with initiatives such as the BIPMed, which gathers members of five CEPIDs (Centers of Research, Innovation, and Dissemination) supported by the FAPESP (São Paulo Research Foundation). The BIPMed is the first public genomic database in Latin America^1^ and has been successful in adding data from healthy individuals that could contribute in future studies of precision medicine.

## Author Contributions

FM was responsible for gathering bibliographic data, writing the draft, editing, and submitting the article to the journal. CB and JR revised the manuscript, made a critical revision, and gave the approval for the final submission.

## Conflict of Interest Statement

The authors declare that the research was conducted in the absence of any commercial or financial relationships that could be construed as a potential conflict of interest.

## Footnotes

1 bipmed.iqm.unicamp.br

## Abbreviations

BIPMed: Brazilian Initiative on Precision Medicine
CEPID: Centros de Pesquisa, Inovação e Difusão
CF: cystic fibrosis
CFTR: cystic fibrosis transmembrane regulator
CRISPR-Cas9: clustered regularly interspaced short palindromic repeats-CRISPR-associated endonuclease 9
FAPESP: Fundação de Amparo à Pesquisa do Estado de São Paulo
OMIM: Online Mendelian Inheritance in Man
TALEN: transcription activator-like effector nuclease
